# Blocking Ocular Sympathetic Activity Inhibits Choroidal Neovascularization

**DOI:** 10.3389/fnins.2021.780841

**Published:** 2022-01-10

**Authors:** Juan Carlos Martinez-Camarillo, Christine K. Spee, Gloria Paulina Trujillo-Sanchez, Anthony Rodriguez, David R. Hinton, Alessandra Giarola, Victor Pikov, Arun Sridhar, Mark S. Humayun, Andrew C. Weitz

**Affiliations:** ^1^USC Roski Eye Institute, Keck School of Medicine, University of Southern California, Los Angeles, CA, United States; ^2^USC Ginsburg Institute for Biomedical Therapeutics, University of Southern California, Los Angeles, CA, United States; ^3^Department of Pathology, Keck School of Medicine, University of Southern California, Los Angeles, CA, United States; ^4^Galvani Bioelectronics, Stevenage, United Kingdom

**Keywords:** wet AMD, internal carotid nerve, choroidal neovascularization, ocular sympathetic activity, laser-induced CNV, β-adrenoreceptor modulation

## Abstract

**Purpose:** To investigate how modulating ocular sympathetic activity affects progression of choroidal neovascularization (CNV), a hallmark feature of wet age-related macular degeneration (AMD).

**Methods:** In the first of two studies, Brown Norway rats underwent laser-induced CNV and were assigned to one of the following groups: daily eye drops of artificial tears (*n* = 10; control group); daily eye drops of the β-adrenoreceptor agonist isoproterenol (*n* = 10); daily eye drops of the β-adrenoreceptor antagonist propranolol (*n* = 10); sympathetic internal carotid nerve (ICN) transection 6 weeks prior to laser-induced CNV (*n* = 10). In the second study, rats underwent laser-induced CNV followed by ICN transection at different time points: immediately after the laser injury (*n* = 6), 7 days after the laser injury (*n* = 6), and sham surgery 7 days after the laser injury (*n* = 6; control group). All animals were euthanized 14 days after laser application. CNV development was quantified with fluorescein angiography and optical coherence tomography (*in vivo*), as well as lesion volume analysis using 3D confocal reconstruction (postmortem). Angiogenic growth factor protein levels in the choroid were measured with ELISA.

**Results:** In the first study, blocking ocular sympathetic activity through pharmacological or surgical manipulation led to a 75% or 70% reduction in CNV lesion volume versus the control group, respectively (*P* < 0.001). Stimulating ocular sympathetic activity with isoproterenol also led to a reduction in lesion volume, but only by 27% versus controls (*P* < 0.05). VEGF protein levels in the choroid were elevated in the three treatment groups (*P* < 0.01). In the second study, fluorescein angiography and CNV lesion volume analysis indicated that surgically removing the ocular sympathetic supply inhibited progression of laser-induced CNV, regardless of whether ICN transection was performed on the same day or 7 days after the laser injury.

**Conclusion:** Surgical and pharmacological block of ocular sympathetic activity can inhibit progression of CNV in a rat model. Therefore, electrical block of ICN activity could be a potential bioelectronic medicine strategy for treating wet AMD.

## Introduction

Age-related macular degeneration (AMD) is among the most common causes of vision loss in developed countries (Campochiaro, 2013). Exudative (wet) AMD is a form of the disease in which an imbalance between angiogenic and antiangiogenic factors, such as vascular endothelial growth factor (VEGF), leads to choroidal neovascularization (CNV) (Van Lookeren Campagne et al., 2014). Anti-VEGF therapies are the gold standard therapy for wet AMD, but these drugs must be injected into the eye every 2–6 weeks (Menon and Walters, 2009).

The sympathetic nervous system may play a role in regulating endpoints related to wet AMD. Sympathetic innervation of the eye originates from the superior cervical ganglion (SCG) (Smith and Reddy, 1990). Prior studies in rat have shown a role for the SCG in regulating choroidal vascularity, with removal of the SCG leading to an increase in vascularization within weeks (Steinle et al., 2002; Steinle and Smith, 2003) accompanied by changes in angiogenic growth factors (Lashbrook and Steinle, 2005; Steinle and Lashbrook, 2006; Wiley et al., 2006). Our group recently demonstrated similar effects through transection of the internal carotid nerve (ICN), a branch of the SCG that is the eye’s only source of sympathetic input (Martinez-Camarillo et al., 2019). We reported that ICN transection led to increased choroidal vascularity and levels of angiogenic factors, including VEGF and tumor necrosis factor-alpha (TNF-α). These results indicate potential involvement of the sympathetic system in CNV and therefore in wet AMD.

The most widely accepted animal model of wet AMD is the laser photocoagulation model (Pennesi et al., 2012). The model works by burning Bruch’s membrane with a laser, which causes growth of new blood vessels from the choroid into the subretinal space (Lambert et al., 2013). This growth is accompanied by upregulation of VEGF (Yi et al., 1997; Wada et al., 1999) and TNF-α (Shi et al., 2006; Jasielska et al., 2010). Maximal changes are observed 1–2 weeks following the laser injury, with involution of the CNV and recovery of the retinal pigment epithelium occurring thereafter (Hoerster et al., 2012; Pennesi et al., 2012). Although the laser-induced CNV animal model was originally developed in non-human primates (Ryan, 1979), rodent models have emerged as the most employed species for neovascular AMD research (Pennesi et al., 2012). While the laser photocoagulation model doesn’t replicate the complete pathophysiology of AMD, it is still the most commonly used animal model for developing wet AMD therapies (Shah et al., 2015; Lin et al., 2019).

Expression of β-adrenergic receptors as part of the sympathetic pathway within the choroid has been described previously (Casini et al., 2014). Propranolol, a non-selective β-adrenoceptor (β-AR) blocker, has been used as an antiangiogenic compound for treating choroidal diseases such as choroidal hemangioma (Thapa and Shields, 2013; O’Bryhim et al., 2019). Prior studies using the laser photocoagulation model in mouse have demonstrated that systemic (Lavine et al., 2013) or intraocular (Nourinia et al., 2015) delivery of propranolol causes a reduction in CNV lesion size. These findings support manipulation of ocular sympathetic activity as a potential therapy for wet AMD. However, they appear to contradict our prior findings that ICN transection (i.e., blocking sympathetic activity) is proangiogenic (Martinez-Camarillo et al., 2019).

The field of bioelectronic medicine has recently emerged with a goal of treating diseases caused by autonomic disbalance (Birmingham et al., 2014). These therapies typically involve electrical stimulation or blocking of autonomic nerves to selectively affect the function of individual organs innervated by those nerves. We have hypothesized that chronic electrical modulation (stimulation or block) of ICN activity can slow, stop, or even reverse progression of wet AMD by normalizing expression of angiogenic growth factors that regulate blood vessel proliferation (Martinez-Camarillo et al., 2019). In the present study, we tested this hypothesis by using pharmacological or surgical manipulation of ocular sympathetic activity as a proxy for ICN electrical modulation and the rat laser photocoagulation model as a proxy for wet AMD. Outcome measures included quantification of CNV development and measurement of choroidal VEGF levels.

## Materials and Methods

### Animals and Study Design

A total of 58 Brown Norway rats, aged postnatal day (P) 100 ± 5 days, were included in two consecutive studies. In the first study, 40 female rats were assigned into one of the following four groups (*n* = 10 per group): (1) laser injury followed by 14 days topical therapy with artificial tears (control group); (2) laser injury followed by 14 days topical therapy of 50 mM isoproterenol eye drop formulation (β-AR agonist group); (3) laser injury followed by 14 days topical therapy with 2% propranolol eye drop formulation (β-AR antagonist group); (4) bilateral ICN transection 6 weeks prior to laser injury followed by 14 days topical therapy with artificial tears (ICNx group). Female animals were used to be consistent with prior studies (Steinle et al., 2002, 2005; Steinle and Smith, 2003; Lashbrook and Steinle, 2005; Steinle and Lashbrook, 2006; Wiley et al., 2006; Martinez-Camarillo et al., 2019). In the second study, 18 male Brown Norway rats were assigned to the following three groups (*n* = 6 per group): (1) bilateral ICN sham surgery 7 days after laser therapy (control group); (2 and 3) bilateral ICN transection immediately after or 7 days after laser therapy. Male animals were used to avoid possible confounding effects from the menstrual cycle on angiogenic growth factor levels. All animals were euthanized 14 days after laser application. CNV development was quantified *in vivo* with fluorescein angiography (FA) and spectral-domain optical coherence tomography (SD-OCT), as well as postmortem with lesion volume analysis using 3D confocal reconstruction. VEGF protein levels in the choroid were measured with ELISA. Table 1 indicates which of these outcome measures were analyzed in each study. Unpaired *t*-tests were used for all statistical comparisons. Investigators who performed data analysis were blinded to the treatment group. All animals received the same anesthesia protocol, which included an intraperitoneal injection of ketamine/xylazine. All experiments were performed in accordance with the University of Southern California Institutional Animal Care and Use Committee (IACUC) approval and guidelines on animal use and with the Association for Research in Vision and Ophthalmology (ARVO) statement for the Use in Ophthalmic and Vision Research.

**TABLE 1:** Analyses performed to track CNV progression following laser injury (day 0).

	Analysis	Time point(s)	Measurement method
Study 1	CNV leakiness	Day 14	FA
	Lesion volume (*ex vivo*)	Postmortem	3D confocal reconstruction
	Choroidal VEGF protein levels	Postmortem	ELISA
Study 2	Lesion volume and edema (*in vivo*)	Days 3, 7, 10, and 14	SD-OCT
	CNV leakiness	Days 3, 7, 10, and 14	FA
	Lesion volume (*ex vivo*)	Postmortem	3D confocal reconstruction

### Internal Carotid Nerve Transection

A subset of animals underwent bilateral transection of the ICN, using a technique previously published by our group (Martinez-Camarillo et al., 2019). This surgical approach selectively disrupts sympathetic supply to the eye, while preserving the other SCG branches. Following transection, the skin incision was closed with a non-absorbable suture (nylon 6-0), and antibiotic ointment was applied. Success of the surgery was verified by monitoring eyelid and eyeball position over the subsequent days (Savastano et al., 2010; Martinez-Camarillo et al., 2019).

### Laser Photocoagulation Injury

With the animals under anesthesia, eye drops were instilled (1% tropicamide and 2.5% phenylephrine HCl) to induce full pupil dilation. Rats were treated with a 532-nm OcuLight GL green diode laser (IRIDEX, Toronto, ON, Canada). Laser settings were: 150–160 mW power, 50 ms duration, and 75 μm diameter. In study 1, half of the rats in each experimental group received 4 burns per eye (one burn per quadrant) and were used for evaluating CNV development with FA scoring and lesion volume analysis using 3D confocal reconstruction (He et al., 2005). The other half received 12 burns in a single eye (one burn per clock hour) and were used for evaluating choroidal VEGF protein levels (Chan et al., 2005) (Twelve burns is considered a blinding procedure, so only one eye could be treated according to animal care guidelines). In study 2, all rats were treated with 4 laser burns per eye. Care was taken to avoid the retinal vessels. Rupture of Bruch’s membrane was confirmed by the presence of a retinal bubble.

### Eye Drops

Following the laser injury, animals in study 1 received daily eye drops between 9:00 and 11:00 am each day for 14 days. Two drops (∼40 μL each) were instilled per eye. Artificial tears were applied in the control and ICNx groups. The β-AR agonist group received 50 mM isoproterenol (Jiang et al., 2010) dissolved in artificial tears, and the β-AR antagonist group received 2% propranolol (Dal Monte et al., 2013) dissolved in artificial tears. Isoproterenol is known to be subject to oxidation, especially at pH levels ≥6.5, which can lead to chemical degradation (Leach et al., 1977). However, the pH of the eye drops could not be adjusted below 6.5 since that would irritate the eyes. In order to mitigate degradation, isoproterenol and propranolol drops were freshly prepared once per week in plastic vials and stored at 4°C. UV spectra of the isoproterenol drops were measured over time to confirm drug stability (Siva et al., 2012).

### Fluorescein Angiography

All animals underwent FA to assess leakage from newly formed vessels resulting from the laser injury. Rats in study 1 received FA 14 days after laser therapy (prior to being euthanized), while rats in study 2 received FA 3, 7, 10, and 14 days after laser injury. With the animals fully anesthetized and their eyes dilated, a 0.01 mL intraperitoneal injection of 10% sodium fluorescein dye was applied. Sequential posterior pole images were taken using a RetCam 3 Retinal Camera (Clarity Medical Systems, Pleasanton, CA, United States) with an 80° lens. The intensity of fluorescein staining in late-phase FA was scored according to an established grading scale (Takehana et al., 1999). Lesions were given a score of 0 (no staining), 1 (slightly stained), 2 (moderately stained), or 3 (strongly stained). The scores of all lesions within each treatment group were averaged. Lesions that scored a 0 were excluded from analysis, since those lesions likely represented laser impacts that did not result in CNV (Lambert et al., 2013).

### 3D Confocal Reconstruction

Animals with 4 burns per eye were used for quantifying CNV lesion volume postmortem. Rats were euthanized at least 4–5 h after FA imaging, in order to allow the fluorescein enough time to clear from the circulatory system. Eyes were enucleated, and the cornea, lens, and retina were removed. The sclera-choroid complex was fixed overnight in 4% formalin at 4°C. Tissue was washed the next day and permeabilized with 0.5% Triton-X for 4 h. The eye cups were blocked with 1% BSA for 2 h and placed in 1:50 fluorescein-labeled GSL I isolectin B4 (endothelial cell and macrophage marker; Vector Laboratories, Burlingame, CA, United States) at 4°C overnight. Samples were washed and mounted on slides with mounting media (VECTASHIELD; Vector Laboratories), while making incisions in the eye cups to flatten them. Flat mounts were visualized using the 10x objective of an UltraVIEW spinning disk confocal microscope (PerkinElmer, Waltham, MA, United States). The image stacks were generated in the z-plane, with the microscope set to excite at 488 nm and to detect at 505–530 nm. Images were processed using the microscope’s software, by closely circumscribing and digitally extracting the fluorescent lesion areas throughout the entire image stack (He et al., 2005). Each extracted lesion was processed through the topography software to generate a digital topographic image representation of the lesion, which was measured to indicate the CNV lesion volume.

### Optical Coherence Tomography

In the second study, SD-OCT was used to monitor CNV progression *in vivo* after laser therapy. OCT imaging was performed with an Envisu Bioptigen system (Leica Microsystems, Wetzlar, Germany), with each lesion imaged using 100 horizontal raster scans spaced 16 μm apart, over an area of 1.6 × 1.6 mm. We used a stereological method (three-dimensional interpretation of two-dimensional cross sections) to reconstruct the OCT images in 3D and calculate lesion size, as described previously (Trujillo-Sanchez et al., 2018).

### Angiogenic Growth Factor Levels

In study 1, animals with 12 laser burns were used for evaluating choroidal VEGF protein levels. Posterior poles were isolated from each laser-treated eye and pooled within each of the four experimental groups. Tissues were homogenized in buffer containing mixed protease inhibitors for protein extraction. Total protein concentration was determined by a Bio-Rad protein assay (Bio-Rad Laboratories, Hercules, CA, United States). VEGF protein in the posterior poles was assessed in triplicate with a VEGF ELISA kit (detection range of 3–500 pg/mL; R&D Systems, Minneapolis, MN, United States) (Chan et al., 2005). Protein concentrations were normalized by the total protein.

## Results

### Study 1

In the first study, we measured the effects of pharmacological or surgical manipulation of ocular sympathetic activity on development of laser-induced CNV. Animals were subjected to laser injury and were split into four groups: (1) daily eye drops of artificial tears (control group); (2) daily eye drops of isoproterenol (β-AR agonist group); (3) daily eye drops of propranolol (β-AR antagonist group); (4) bilateral ICN transection 6 weeks prior to the laser injury, followed by daily eye drops of artificial tears (ICNx group). The 6-week delay before the laser injury was chosen to be consistent with prior studies that assessed effects of sympathetic denervation on choroidal vascularity and related measures (Steinle et al., 2002; Steinle and Smith, 2003; Steinle and Lashbrook, 2006; Martinez-Camarillo et al., 2019). Animals were euthanized 14 days after laser application. As described in Table 1 and section “Materials and Methods,” outcome measures included CNV leakiness (*in vivo*), CNV lesion volume (*ex vivo*), and choroidal VEGF protein levels.

#### Fluorescein Angiography

**FIGURE 1:**
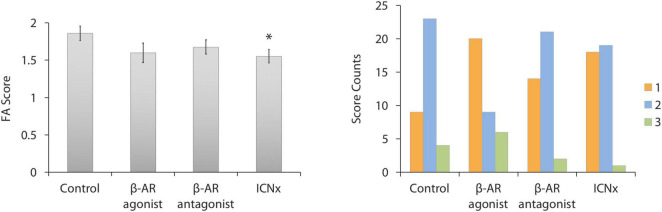
FA scores 14 days after laser injury in each experimental group (*n* ≥ 35 lesions per group from five animals per group). Four laser burns were made per eye. **(Left)** Average lesion score in each group. Scores in the ICN transection group were significantly lower than scores in the control group (*P < 0.05). Error bars indicate SEM. **(Right)** Histogram showing the distribution of FA scores in each group.

Leakiness of CNV lesions was assessed with FA scoring, 14 days after laser therapy. Average FA scores for all groups fell between 1.5 and 2.0, indicating moderate staining (Figure 1). Lesions in rats receiving daily β-AR eye drops (propranolol or isoproterenol) were slightly less leaky than lesions in the control group; however, scores were not significantly different among these groups (*P* > 0.05). In rats that underwent ICN transection 6 weeks prior to laser injury, FA scores were 17% lower than scores in the control group (*P* = 0.02).

#### 3D Confocal Reconstruction

**FIGURE 2:**
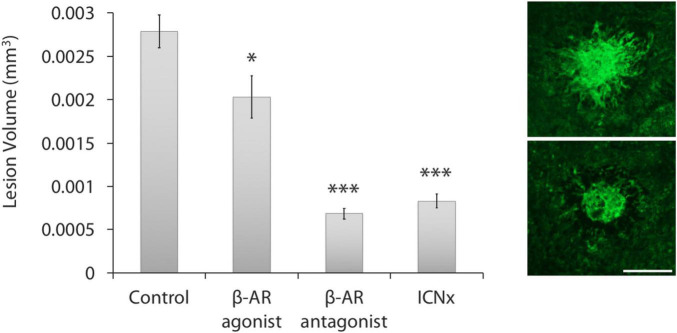
β-AR modulation leads to smaller laser-induced CNV lesions. **(Left)** All three treatment groups had statistically smaller lesions than the control group (*n* ≥ 30 lesions per group from five animals per group; ^***^P < 0.001; *P < 0.05). Lesions in the propranolol and ICN transection groups were statistically similar in size (*P* = 0.16). Error bars represent SEM. **(Right)** Images of CNV membranes, stained with FITC-labeled isolectin-B4, showing representative lesions from the control group (top) and ICN transection group (bottom). Scale bar = 200 μm.

Confocal analysis of lesion volumes revealed smaller lesions in the three treatment groups than in the control group (Figure 2). Blocking β-AR activity with propranolol or through ICN transection led to reductions in lesion volume by 75 and 70%, respectively (*P* < 0.001). Application of the β-AR agonist isoproterenol also reduced lesion volume versus the control group, but by only 27% (*P* < 0.05).

#### Angiogenic Growth Factor Levels

**FIGURE 3:**
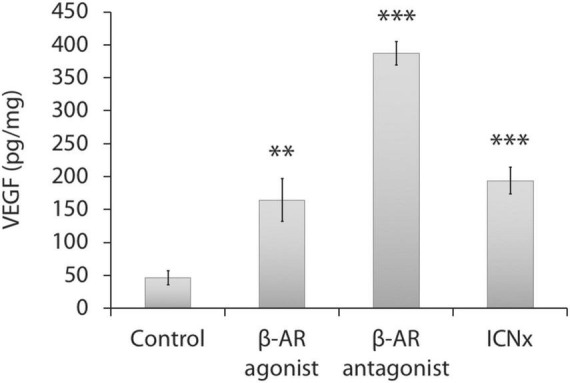
Choroidal VEGF protein levels 14 days after laser injury in each experimental group (*n* = 5 animals per group). Twelve laser burns were made in a single eye. VEGF levels in the treatment groups were significantly higher than levels in the control group. Units represent VEGF protein normalized to total protein. Error bars represent SD. ^***^P < 0.001; ^**^P < 0.01.

ELISA testing indicated elevated choroidal VEGF protein levels in the three treatment groups versus the control group (*P* < 0.01; Figure 3). VEGF levels in the control group were relatively low (Martinez-Camarillo et al., 2019), suggesting a return to baseline in this group.

### Study 2

The second study was designed to further investigate the effects of ICN transection in the rat laser-induced CNV model. Results from study 1 revealed that blocking ocular sympathetic activity in this model inhibited progression of laser-induced CNV (see Figures 1, 2), supporting ICN block as a potential therapy for wet AMD. However, ICN transection was performed 6 weeks prior to the laser injury. A therapy for wet AMD would not commence until after a patient presents with CNV; therefore, a better animal model of the clinical situation would be to perform ICN transection *after* the laser injury. This was the purpose of study 2.

Rats were subjected to laser injury and split into three groups: (1) bilateral ICN sham surgery 7 days after the laser injury (control group); (2) bilateral ICN transection immediately after the laser injury (ICNx_0_ group); (3) bilateral ICN transection 7 days after the laser injury (ICNx_7_ group). Animals were euthanized 14 days after laser application. Outcome measures included CNV leakiness (*in vivo*) and CNV volume (*in vivo* and *ex vivo*), as described in Table 1 and section “Materials and Methods.”

#### Fluorescein Angiography

**FIGURE 4:**
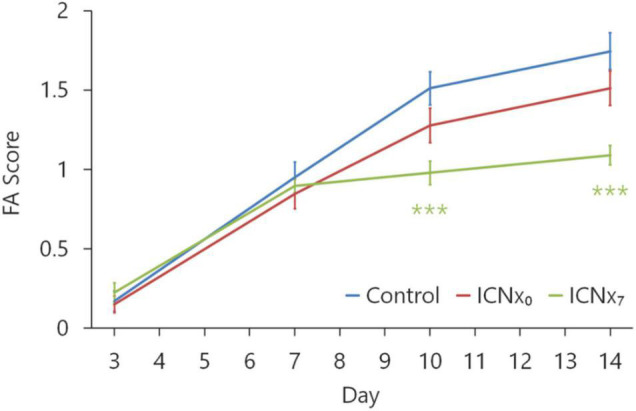
FA scores at 3, 7, 10, and 14 days after laser injury in each experimental group (*n* ≥ 45 lesions per group from six animals per group). Four laser burns were made per eye. Beginning 10 days after laser treatment, scores in the animals that underwent ICN transection on day 7 were significantly lower than scores in the animals that received sham surgery (***P < 0.001). Error bars indicate SEM.

Figure 4 shows the FA scores in each experimental group at 3, 7, 10, and 14 days after the laser injury. Scores in all groups were statistically similar on days 3 and 7, with the average score increasing from ∼0.2 to ∼0.9 over this time period. Scores in each group increased again on days 10 and 14, indicating steady CNV development throughout the 2-week monitoring period. On days 10 and 14, FA scores in both ICNx groups were lower than those of the control group, with the ICNx_7_ group exhibiting the lowest average scores. The average FA score in the ICNx_7_ group increased from ∼0.9 to ∼1.1 between days 7 and 14, signifying limited CNV progression over this time period.

#### Lesion Volume Analysis

**FIGURE 5:**
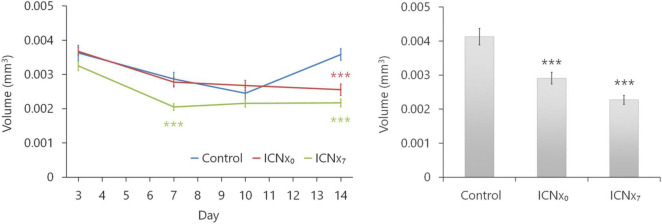
ICN transection leads to smaller laser-induced CNV lesions. Lesion volumes were measured with SD-OCT at 3, 7, 10, and 14 days after laser treatment (left). Following euthanasia on day 14, volumes were measured *ex vivo* with confocal microscopy (right). By day 14, animals that underwent ICN transection had statistically smaller lesions than the animals that received sham surgery (*n* ≥ 44 lesions per group from six animals per group; ***P < 0.001). This was true regardless of whether ICN transection was performed immediately after or 7 days after laser therapy. Error bars represent SEM.

Figure 5 summarizes the results from the OCT and confocal lesion volume measurements. Lesions in all three groups shrank between days 3 and 7 after the laser injury, due to resolution of edema during this time period. Between days 7 and 10, lesion sizes remained relatively stable. Average lesion sizes in all groups were statistically similar through day 10, with just one exception (see Figure 5, *left*). By day 14, however, lesions in the control group had grown, while lesion sizes in the two ICNx groups remained stable. Both OCT and confocal imaging revealed significantly smaller lesion sizes in the ICNx groups versus the control group on day 14 (*P* < 0.001). According to the confocal measurements, average lesion volume in the ICNx_0_ and ICNx_7_ groups was 30 and 45% smaller than that of the control group, respectively.

## Discussion

Outcomes from our two studies indicate a direct role for the sympathetic nervous system, and the β-AR receptors in particular, in regulating the development of laser-induced CNV. In the first study, inhibiting sympathetic activity by either pharmacological or surgical manipulation led to reductions in lesion volume by 70–75%. In agreement with the FA data (see Figure 1), these results suggest that blocking ocular sympathetic activity generated an anti-angiogenic response. Unexpectedly, application of the β-AR agonist isoproterenol also led to a reduction in lesion volume versus the control group, but only by 27%. This may be attributed to β-AR desensitization or downregulation caused by prolonged application of isoproterenol, as reported by others (Gonzalez-Brito et al., 1988; Gambarana et al., 1991; Brouri et al., 2002; Dal Monte et al., 2012).

Though no studies have investigated the effect of β-AR agonists on the progression of laser-induced CNV, several studies in mice have reported that systemic or intraocular delivery of β-AR antagonists causes a reduction in CNV lesion size (Lavine et al., 2013, 2017; Nourinia et al., 2015; Omri et al., 2019). Lavine et al. (2013) measured lesion areas (as opposed to volumes) 14 days after laser photocoagulation and found that daily intraperitoneal injection of propranolol (20 mg/kg/day) led to a 50% reduction in lesion size. Omri et al. (2019) also treated mice with daily administration of intraperitoneal propranolol (6 mg/kg/day) and observed ∼70% reduced lesion areas after 14 days. Nourinia et al. (2015) measured lesion areas 28 days after laser photocoagulation and found that a single intravitreal injection of propranolol (0.3 μg) at the time of laser application led to a 79% reduction in lesion size, similar to what we observed. Lavine et al. (2017) tested effects of a single intravitreal injection of the β_2_-AR antagonist ICI 118,551 at the time of laser application and found a 35% reduction in lesion area after 14 days, indicating that the anti-angiogenic effects reported in these studies are at least partially due to β_2_ receptor blockade (as opposed to other β-AR subtypes).

To further demonstrate the inhibitory effect of delayed ocular sympathetic block on laser-induced CNV in rats, the second study showed that surgical ICN transection led to smaller CNV lesion sizes, even when transection was performed at 0 and 7 days after the laser injury. Unexpectedly, we found that ICN transection 7 days after the laser injury was more effective than immediately after the laser injury (see Figures 4, 5). This finding may arise from use of relatively small sample sizes (*n* = 6 animals per group). In support of this hypothesis, it would be expected that 7 days after the laser injury, CNV lesions in the ICNx_7_ group (prior to undergoing surgery) would be similar in size to lesions in the control group. However, as shown in Figure 5, ICNx_7_ lesions were smaller than control group lesions on day 7. Nevertheless, the FA and lesion volume results from both studies indicate that surgically removing the ocular sympathetic supply inhibits progression of laser-induced CNV. Surgical ICN block was more effective when performed 6 weeks prior to laser injury versus after laser injury (70% reduction in lesion volumes versus 30–45% reduction, respectively). For comparison, a mouse study with the FDA-approved anti-VEGF agent aflibercept (Eylea; VEGF-TRAP_R1R2_) demonstrated that a single intravitreal injection of the drug at the time of laser application led to a ∼30% reduction in lesion size after 14 days (Saishin et al., 2003).

Given our finding that pharmacological and surgical block of ocular sympathetic activity inhibited CNV progression, it would be expected that these interventions would also cause reduced choroidal VEGF levels. However, we found just the opposite; choroidal VEGF was elevated in the treatment groups (see Figure 3). There are a couple potential explanations for this surprising result: First, VEGF was measured 14 days after the laser injury, which may not have been the appropriate time point [for example, systemic VEGF levels in mouse peak 7 days after laser injury and return to baseline after 14 days (Kase et al., 2010)]. Second, VEGF levels are affected by several factors including inflammation, ischemia, and hypoxia (Ramakrishnan et al., 2014); it is possible that drug administration and/or ICN transection surgery caused these side effects.

Our observation that blocking ocular sympathetic activity in the laser-induced CNV model is anti-angiogenic appears to contradict our prior finding that ICN transection in naive rats (not subjected to laser injury) causes increased choroidal vascularity after 6 weeks, as measured by histomorphometry (Martinez-Camarillo et al., 2019). One possible explanation, as proposed previously, is that this increased vascularity may have been indirectly caused by a long-term vasodilation due to a loss of sympathetic tone (Martinez-Camarillo et al., 2019). Another possibility is that the contradictory findings arise from use of different experimental models and rat strains: naive Sprague Dawley (albino) in our prior study and laser-treated Brown Norway (pigmented) in the present studies (The laser-induced CNV model requires pigmented animals, since pigment is needed for absorbing the laser energy to create a burn). Yet another possibility is that sympathetic activity plays different roles in the intact versus leaky blood vessels, which is supported by a pro-angiogenic sympathetic role in tumor neovascularization (Mulcrone et al., 2017; Hanns et al., 2019; Kamiya et al., 2019; Stavropoulos et al., 2020). In a comprehensive review of the role of the β-adrenergic system on ocular neovascularization, Casini et al. (2014) concluded that “in different experimental models, a decrease of the β-adrenergic function may result either in reduction or in exacerbation of the vascular changes, thus suggesting possible dual effects of β-AR modulation depending on the experimental setting.” Because the laser-induced CNV model is the gold standard for testing new treatments for wet AMD (Pennesi et al., 2012), future studies should focus on this model.

In summary, our results demonstrate that blocking ocular sympathetic activity inhibits CNV. Even when ICN transection was performed 1 week after laser injury, inhibition of CNV progression was still observed. This suggests that electrical blocking of ICN activity could be an effective bioelectronic medicine strategy for treating wet AMD.

## Data Availability Statement

The raw data supporting the conclusions of this article will be made available by the authors, without undue reservation.

## Ethics Statement

The animal study was reviewed and approved by the USC Institutional Animal Care and Use Committee.

## Author Contributions

JCM-C, MH, DH, and AW contributed to conception and design of the study. JCM-C, CS, GT-S, AR, AG, VP, AS, and AW conducted the experiments and organized the database. AW performed the statistical analysis. JCM-C wrote the first draft of the manuscript. CS, AR, and AW wrote sections of the manuscript. All authors contributed to manuscript revision, read, and approved the submitted version.

## Conflict of Interest

AG, VP, and AS were employed by the company Galvani Bioelectronics. The remaining authors declare that the research was conducted in the absence of any commercial or financial relationships that could be construed as a potential conflict of interest. The authors declare that this study received funding from Galvani Bioelectronic. The funder had the following involvement in the study: Study design and interpretation of data.

## Publisher’s Note

All claims expressed in this article are solely those of the authors and do not necessarily represent those of their affiliated organizations, or those of the publisher, the editors and the reviewers. Any product that may be evaluated in this article, or claim that may be made by its manufacturer, is not guaranteed or endorsed by the publisher.
